# Long-Term Frequent Use of Non-Steroidal Anti-Inflammatory Drugs Might Protect Patients with Ankylosing Spondylitis from Cardiovascular Diseases: A Nationwide Case-Control Study

**DOI:** 10.1371/journal.pone.0126347

**Published:** 2015-05-13

**Authors:** Wen-Chan Tsai, Tsan-Teng Ou, Jeng-Hsien Yen, Cheng-Chin Wu, Yi-Ching Tung

**Affiliations:** 1 Department of Internal Medicine, Kaohsiung Medical University Hospital, Kaohsiung, Taiwan; 2 Department of Public Health and Environmental Medicine, College of Medicine, Kaohsiung Medical University, Kaohsiung, Taiwan; University of Hull, UNITED KINGDOM

## Abstract

The objective of this case-control study was to investigate the risk of cardiovascular disease (CVD) following non-steroidal anti-inflammatory drug (NSAID) use in patients with ankylosing spondylitis (AS). A total of 10,763 new AS patients were identified from the National Taiwan Health Insurance claims database during the period from 1997 to 2008. In all, 421 AS patients with CVD were recruited as cases, and up to 2-fold as many sex- and age-matched controls were selected. Logistic regression models were used to estimate the odds ratio (OR) between NSAID use and CVD incidence. The medication possession rate (MPR) was used to evaluate NSAID exposure during the study period. AS patients had increased risk of CVD (OR, 1.68; 95% confidence interval (CI), 1.57 to 1.80). Among frequent (MPR≥80%) COX II users, the risks for all types of CVD were ten times lower than those among non-users at 24 months (OR, 0.08; 95% CI, 0.01 to 0.92). Among frequent NSAID users, the risks of major adverse cardiac event (MACE) were significantly lower at 12 months (OR, 0.23; 95% CI, 0.07 to 0.76)—a trend showing that longer exposure correlated with lower risk. Regarding non-frequent NSAID users (MPR<80%), short-term exposure did carry higher risk (for 6 months: OR, 1.41; 95% CI, 1.07 to 1.86), but after 12 months, the risk no longer existed. We conclude that long-term frequent use of NSAIDs might protect AS patients from CVD; however, NSAIDs still carried higher short-term risk in the non-frequent users.

## Introduction

Non-steroidal anti-inflammatory drugs (NSAIDs) are among the most commonly prescribed drugs in the world. For many musculoskeletal conditions and inflammatory diseases, toothache and even dysmenorrhea, NSAIDs are the drugs of choice [1,2]. Over the past 50 years, traditional NSAIDs have become notorious for their GI complications [3–5]. In the 1990s, a new class of NSAIDs that were designed for specific inhibition of cyclooxygenase II (COX II) was launched and proved to reduce the risk of GI complications [6,7]. Unfortunately, a new concern arose for COX II inhibitors: increased cardiovascular risk. In the Vioxx Gastrointestinal Outcomes Research (VIGOR) study, rofecoxib was shown to be associated with a much higher risk of myocardial infarction than the comparator drug (naproxen) [7]. Furthermore, data from clinical trials [8–10], epidemiological studies [11,12] and a meta-analysis [13] indicated that both traditional NSAIDs and COX II inhibitors increase the occurrence of cardiovascular events. In the latest meta-analysis, all NSAIDs increased hospitalization for heart failure, and coxibs and diclofenac increased the frequency of major vascular events [14]. Panic has spread to both patients and physicians, certain publications advocate that even a low event rate must be taken seriously, given that NSAIDs are prescribed primarily for symptom relief. It is well known that autoimmune/auto-inflammatory diseases are associated with an increased risk of cardiovascular disease (CVD) [15,16]. Rheumatoid arthritis (RA) is reported to be associated with a 2- to 3-fold increased risk [17]; systemic lupus erythematosus, with an at least 2-fold increased risk [18,19]; and ankylosing spondylitis (AS), with a 1.3- to 2.2-fold increased risk [20,21]. Autoimmune/auto-inflammatory diseases and atherosclerosis have been found to share similar inflammatory processes, suggesting that mechanisms of auto-inflammatory cascades contribute to the excessive cardiovascular risk of autoimmune/auto-inflammatory diseases [22]. Given that an inflammatory process mediates atherosclerosis, it has been suggested that the medications used to control inflammation may potentially reduce the cardiovascular risk of autoimmune/auto-inflammatory diseases. Tumor necrosis factor (TNF) inhibitors are widely used for the treatment of RA due to their strong anti-inflammatory reaction. Several studies have revealed that TNF inhibitors are effective in reducing cardiovascular risks [23–25]. In one study, Bili et al. found that the use of anti-TNF was associated with a 55% reduction in the risk of developing coronary artery disease in an incident cohort of RA patients and that this risk decreased further with prolonged use [26].

Theoretically, mediated by their anti-inflammatory effects, the use of NSAIDs can reduce the cardiovascular risk of autoimmune/auto-inflammatory diseases. However, NSAID use is known to increase cardiovascular risk in the general population. It would be very interesting to know whether the level of NSAID use is pro-CVD or anti-CVD. In this study, we chose patients with AS as the study population to investigate the impact of NSAID use on the risk of cardiovascular events.

## Methods

### Data Sources

The Taiwan National Health Insurance (NHI) database covers health data from 99% of the 23 million inhabitants of Taiwan. This database includes disease diagnosis, hospital admissions, outpatient visits, and prescriptions. The database has been released to researchers in an electronically encrypted form since 1999. The large sample size and the high quality of diagnosis recording, according to the coding system of the International Classification of Diseases—Ninth Revision, Clinical Modification (ICD-9-CM), have ensured that this dataset provides the opportunity to estimate the incidence of CVD in both the general populations and AS patients with different levels of NSAID exposure. The study protocol was approved by the Kaohsiung Medical University Hospital Ethics Committee. The study protocol was approved by the Kaohsiung Medical University Hospital Ethics Committee. Because patient records/information was anonymized and de-identified prior to analysis, informed consent is not required.

### AS Cohort

The records of all patients who were diagnosed with AS using the ICD-9-CM (code 7200) and logged in the claims database between January 1, 1997, and December 31, 2008, were retrieved. In Taiwan, patients who fulfill the 1984 modified New York criteria for AS are defined in NHI database as AS. We recruited those AS patients who had at least two service claims or either ambulatory or inpatient care for further confirmation of their diagnosis. Clinical characteristics included information on overall comorbidity at the time of data mining, which was assessed by computing the Charlson comorbidity index (CCI) [27]. The index applies to 17 disease categories whose scores are totaled to obtain an overall score for each patient.

**Fig 1:**
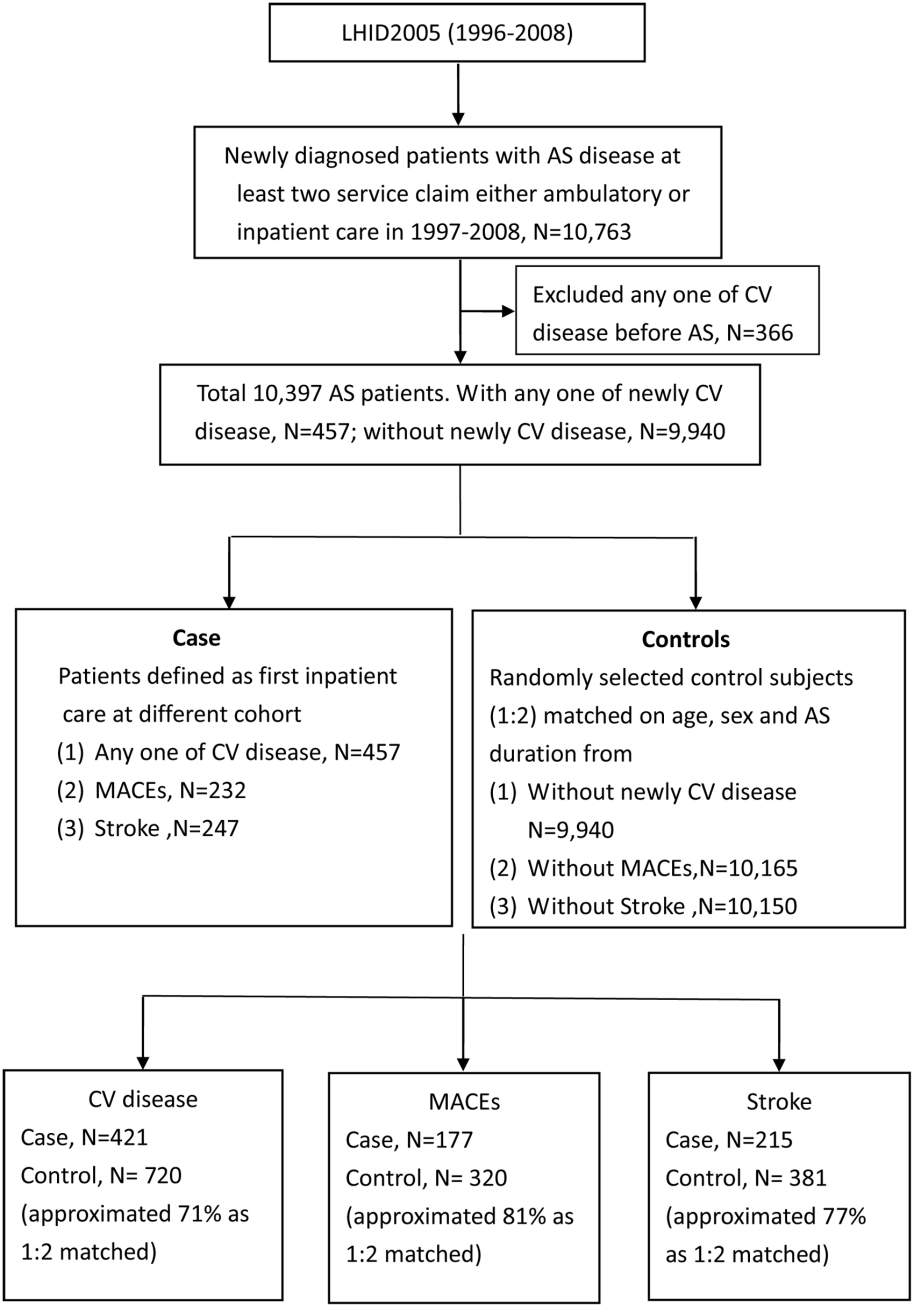
Consort diagram. Abbreviations: AS, ankylosing spondylitis; CV disease, cardiovascular disease; MACEs, major cardiovascular events; LHID, longitudinal health insurance database.

A total of 10,763 patients were selected by the above definition. We excluded 366 patients who had any CVD before the diagnosis of AS. As a result, the study cohort encompassed 10,397 patients. Among them, 457 patients who had newly onset CVD were included as cases. The other 9,940 patients served as the control group. For further analysis of the risk of stroke and major adverse cardiac events (MACEs), 247 and 232 patients who had stroke and MACEs, respectively, were included as cases. In total, 10,150 and 10,165 patients who did not have stroke or MACEs, respectively, were selected as controls. Risk-set sampling matched by sex, age (within 5 years), AS duration at the onset of CVD, and the frequency of NSAID use was used to select controls in the AS cohort. Up to 2 controls were selected for each case (Fig 1).

### NSAID Treatment

All of the NSAIDs prescribed in the claims database were further classified into non-selective NSAIDs and specific COX II inhibitors. We chose the medication possession rate (MPR) as a tool to assess the frequency of NSAID exposure. Three groups were classified: non-user, MPR<80% and MPR≥80%. The durations of drug exposure were defined as 3 months, 6 months, 12 months, 24 months and 36 months.

### Outcomes

The combined endpoints of stroke (ICD-9 430–438), MACEs (acute coronary syndrome: ICD-9 410–410.9, heart failure: ICD-9 428.0–428.9, cerebrovascular accident, stroke: ICD-9 430–438) and any CVD (ICD-9 410–414, 425–426, 427.3, 428, 430–438, 443, 785.4, 444.2) were used as outcomes of NSAID exposure.

### Statistical Analysis

In this study, conditional logistic regression was used to estimate the crude and adjusted odds ratios (ORs) and 95% confidence interval (CI) for the risk of CVD associated with NSAID use. Potential risk factors, including, sex, age, CCI, AS disease duration and other drugs used, were incorporated into the models. The frequency of NSAID exposure, which was assessed using the MPR, was included as a time-dependent variable. CIs were set to 95%, and a two-tailed p value less than 0.05 was considered significant.

## Results

A total of 10,763 patients were recruited from the claims database. Compared with normal populations, the AS patients had an increased risk of CVD. The crude OR was 1.68 (95% CI, 1.57 to 1.80; p<0.0001). After exclusion of 366 patients with CVD onset before the diagnosis of AS, 10,397 patients were selected for further analysis. The baseline characteristics of the AS patients are reported in Table 1. More than 70% of patients were aged less than 55 years old. As shown in Table 1, approximately 53% of patients developed CVD more than 3 years after the diagnosis of AS.

**Table 1:** Baseline characteristics of AS patients and duration from diagnosis to the onset of CV disease.

AS patients (N = 10,397)			AS patients with CV disease (N = 457)	
Characteristics		N (%)	Duration (year)	N (%)
Sex	Female	5,705 (54.87)	<1	61 (13.35)
	Male	4,692 (45.13)	1–2	86 (18.82)
Age	<35	3,027 (29.11)	2–3	66 (14.44)
	35–45	2,082 (20.03)	3–4	50 (10.94)
	45–55	2,336 (22.47)	>4	195 (42.45)
	55–65	1,411 (13.57)		
	>65	1,541 (14.82)		

Abbreviation: AS, ankylosing spondylitis; CV, cardiovascular

**Fig 2:**
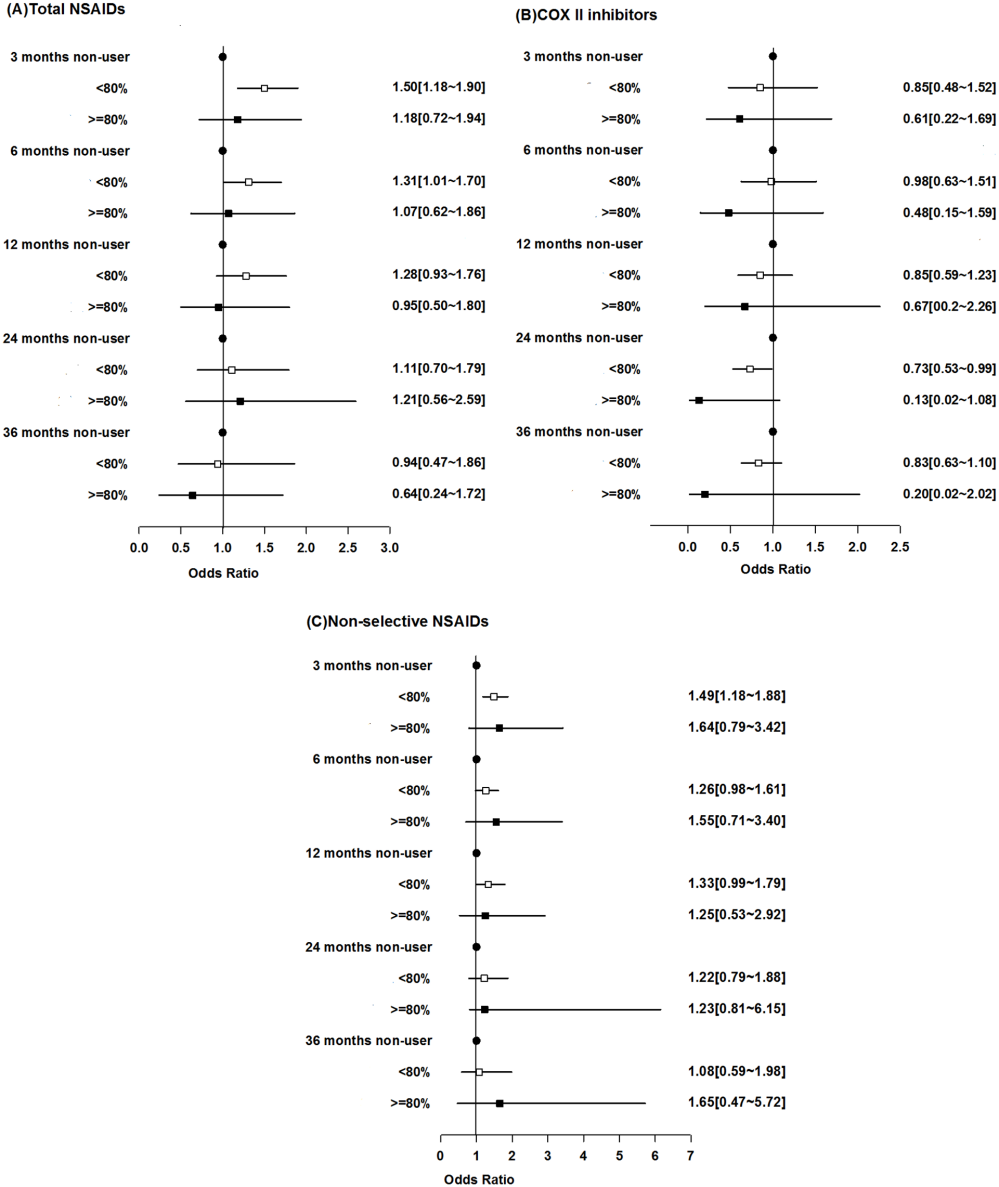
Risks of all types of cardiovascular diseases associated with non-steroidal anti-inflammatory drugs (NSAIDs). (A) Total NSAIDs, (B) specific cyclooxygenase II (COX II) inhibitors, (C) non-selective NSAIDs. NSAID exposure was categorized into non-users, MPR<80% and MPR≥80%. The data were adjusted by the CCI.

**Fig 3:**
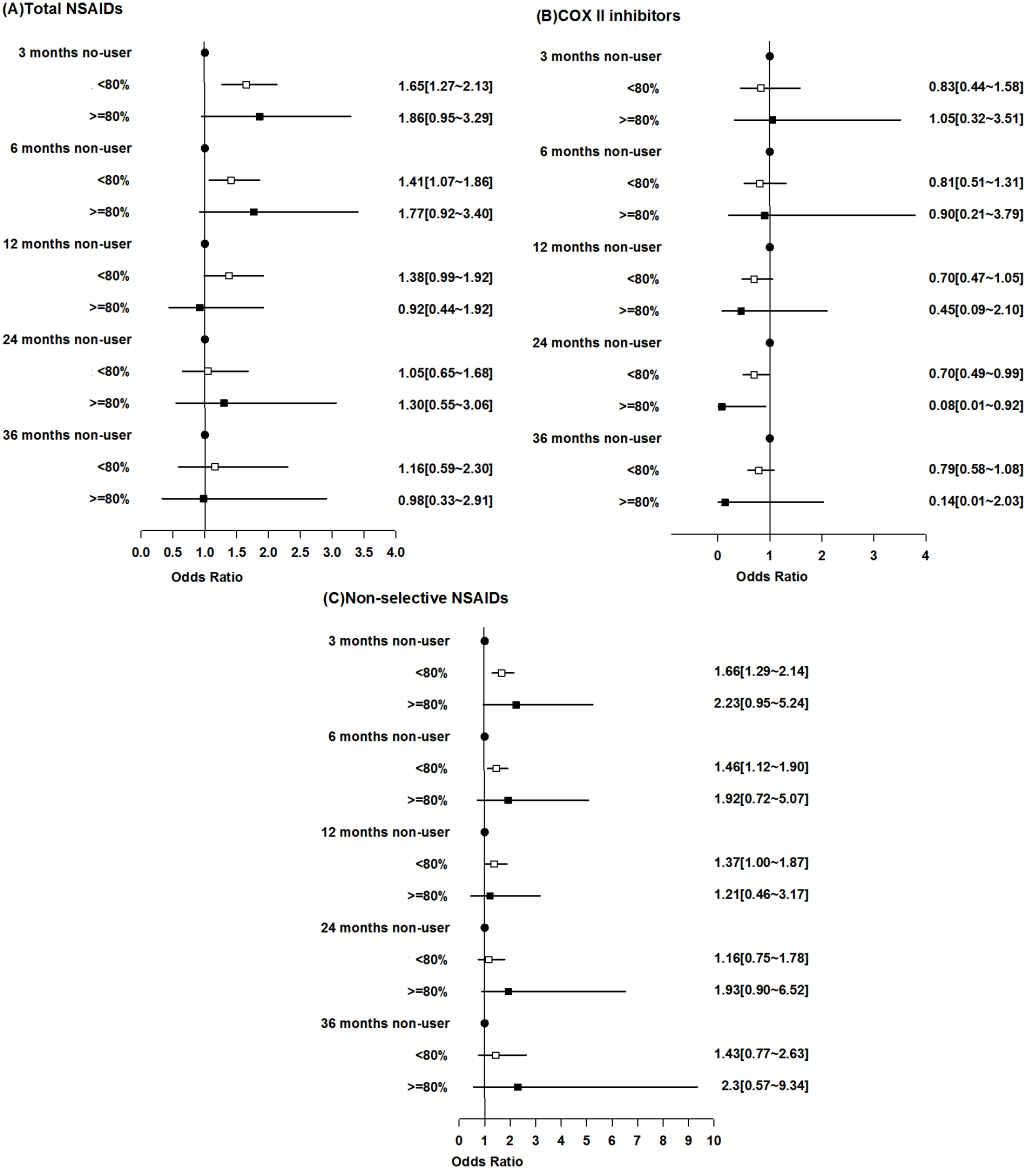
Risks of all types of cardiovascular diseases associated with non-steroidal anti-inflammatory drugs (NSAIDs). (A) Total NSAIDs, (B) specific cyclooxygenase II (COX II) inhibitors, (C) non-selective NSAIDs. NSAID exposure was categorized into non-users, MPR<80% and MPR≥80%. The data were adjusted by the CCI and other drugs used.

For all types of CVD as an endpoint, a total of 421 incident cases and 720 age-, sex- and disease duration-matched controls were identified. After adjustment for the CCI, among patients who were frequent NSAID users (MPR≥80%), there was no statistically significant increase in CVD risk compared with that among non-users at any time point after the initiation of NSAID. Moreover, there was a trend showing that the longer the exposure was, the lower the risk was. For non-frequent NSAID users (MPR<80), a statistically significant increase in CVD risk was found with short-term exposure (at 3 months: OR, 1.50; 95% CI, 1.18 to 1.90; p = 0.001 and at 6 months: OR, 1.31; 95% CI, 1.01 to 1.70; p = 0.0412), but after 12 months, a significant risk was no longer found (Fig 2A). After further adjustment for other drugs used, the results were similar (at 3 months: OR, 1.65; 95% CI, 1.27 to 2.13; p = 0.0002 and at 6 months: OR, 1.41; 95% CI, 1.07 to 1.86; p = 0.0137) (Fig 3A). We further stratified all NSAIDs into two groups: COX II inhibitors and non-selective NSAIDs. Compared with NSAID non-users, COX II users did not have an increased risk of any type of CVD. Instead, after adjustment for CCI and drug, among patients who were frequent COX II user for 24 months, the risks for all types of CVD were ten times lower than that among non-user (OR, 0.08; 95% CI, 0.01 to 0.92; p = 0.042). Even for those non-frequent users, the risk was significantly lower compared with that among non-user (OR, 0.70; 95% CI, 0.49 to 0.99; p = 0.043) (Fig 3B). There was also a trend showing that the longer the use was, the lower the risk was (Fig 2B and Fig 3B). For non-frequent non-selective NSAID users, a mild increase in CVD was found at 3 months (OR, 1.49; 95% CI, 1.18 to 1.88; p = 0.001) (Fig 2C). Similar results were found at 3 months (OR, 1.66; 95% CI, 1.29 to 2.14; p<0.001) after adjustment for other drugs (Fig 3C).

For MACEs as an endpoint, a total of 177 incident cases and 320 age-, sex- and disease duration-matched controls were identified. After adjustment for the CCI, no any NSAID user had a statistically significant increase in MACE risk compared with non-users, no matter for how long and how frequently they were exposed to NSAIDs (Table 2). After further adjustment for drugs, there was significant lower risk for those frequent users at 12 months (OR, 0.23; 95% CI, 0.07 to 0.76; p = 0.016) (S1 Table).

**Table 2:** Risk of MACEs associated with NSAIDs in patients with AS stratified by frequency of exposure and types of NSAIDs adjust for Charlson comorbility index.

Total NSAIDs					COX-II					Non-selective NSAIDs				
		OR	95%CI	P-value			OR	95%CI	P-value			OR	95%CI	P-value
3 months	Non-user	1	-	-	3 months	Non-user	1	-	-	3 months	Non-user	1	-	-
	<80%	1.21	0.87–1.70	0.259		<80%	0.56	0.26–1.25	0.1576		<80%	1.36	0.98–1.90	0.0669
	≥80%	0.78	0.37–1.67	0.5227		≥80%	0.38	0.07–2.18	0.275		≥80%	0.86	0.29–2.52	0.7801
6 months	Non-user	1	-	-	6 months	Non-user	1	-	-	6 months	Non-user	1	-	.
	<80%	1.13	0.79–1.61	0.5125		<80%	0.74	0.40–1.35	0.3218		<80%	1.2	0.85–1.69	0.29
	≥80%	0.81	0.38–1.76	0.5995		≥80%	0.15	0.02–1.45	0.1014		≥80%	1.21	0.44–3.31	0.7155
12 months	Non-user	1	-	-	12 months	Non-user	1	-	-	12 months	Non-user	1	-	-
	<80%	0.85	0.55–1.31	0.4572		<80%	0.61	0.37–1.02	0.0582		<80%	1.03	0.68–1.54	0.9023
	≥80%	0.48	0.19–1.22	0.1252		≥80%	0.43	0.06–3.15	0.4084		≥80%	0.61	0.17–2.20	0.4536
24 months	Non-user	1	-	-	24 months	Non-user	1	-	-	24 months	Non-user	1	-	-
	<80%	0.92	0.48–1.76	0.8022		<80%	0.68	0.44–1.05	0.0801		<80%	0.89	0.50–1.58	0.6893
	≥80%	0.5	0.16–1.56	0.2317		≥80%	1.41	0.08–24.67	0.8122		≥80%	0.7	0.13–3.70	0.6704
36 months	Non-user	1	-	-	36 months	Non-user	1	-	-	36 months	Non-user	1	-	-
	<80%	0.95	0.34–2.69	0.9292		<80%	0.78	0.52–1.15	0.2013		<80%	1.1	0.47–2.53	0.8309
	≥80%	0.52	0.13–2.10	0.3613		≥80%	1.43	0.08–25.09	0.8072		≥80%	1.38	0.23–8.45	0.7263

Abbreviation: NSAIDs, non-steroidal anti-inflammatory drugs; Total NSAID, include COX-II inhibitors and non-selective NSAIDs; COX-II, cyclooxygenase II inhibitors; MACEs, major adverse cardiac events

For stroke as an endpoint, a total of 215 incident cases and 381 age-, sex- and disease duration-matched controls were identified. After adjustment for the CCI, only non-frequent non-selective NSAID users had a statistically significant increase in stroke risk compared with non-users at 3 months (OR, 1.44; 95% CI, 1.00 to 2.07; p = 0.049) (Table 3), but after further adjustment for the drugs used, a significant increase in stroke risk was no longer found (S2 Table).

**Table 3:** Risk of stroke associated with NSAIDs in patients with AS stratified by frequency of exposure and types of NSAIDs adjust for Charlson comorbility index.

Total NSAIDs					COX-II					Non-selective NSAIDs				
		OR	95%CI	P-value			OR	95%CI	P-value			OR	95%CI	P-value
3 months	Non-user	1	-	-	3 months	Non-user	1	-	-	3 months	Non-user	1	-	-
	<80%	1.35	0.93–1.95	0.1128		<80%	0.87	0.36–2.12	0.7655		<80%	1.44	1.00–2.07	**0.0494**
	≥80%	0.9	0.39–2.05	0.796		≥80%	0.38	0.07–2.15	0.2749		≥80%	0.6	0.16–2.22	0.447
6 months	Non-user	1	-	-	6 months	Non-user	1	-	-	6 months	Non-user	1	-	-
	<80%	1.2	0.82–1.77	0.351		<80%	0.99	0.51–1.93	0.9747		<80%	1.26	0.87–1.83	0.226
	≥80%	0.97	0.43–2.20	0.939		≥80%	0.18	0.02–1.85	0.1495		≥80%	1.76	0.54–5.73	0.3459
12 months	Non-user	1	-	-	12 months	Non-user	1	-	-	12 months	Non-user	1	-	-
	<80%	1	0.63–1.59	0.9999		<80%	0.84	0.49–1.45	0.5321		<80%	1.21	0.78–1.87	0.3959
	≥80%	0.61	0.23–1.63	0.3214		≥80%	0.29	0.04–1.84	0.1877		≥80%	1.27	0.29–5.65	0.752
24 months	Non-user	1	-	-	24 months	Non-user	1	-	-	24 months	Non-user	1	-	-
	<80%	1.04	0.51–2.10	0.9231		<80%	0.79	0.49–1.26	0.3158		<80%	1	0.54–1.86	0.9933
	> = 80%	0.72	0.22–2.36	0.5864		≥80%	0.36	0.03–3.75	0.3894		≥80%	1.07	0.19–6.16	0.9407
36 months	Non-user	1	-	-	36 months	Non-user	1	-	-	36 months	Non- user	1	-	-
	<80%	0.98	0.30–3.23	0.9755		<80%	0.97	0.63–1.49	0.8947		<80%	1.3	0.50–3.36	0.5905
	≥80%	0.55	0.12–2.60	0.4537		≥80%	0.14	0.09–24.53	0.7907		≥80%	1.36	0.21–8.97	0.7474

Abbreviation: NSAIDs, non-steroidal anti-inflammatory drugs; Total NSAID, include COX-II inhibitors and non-selective NSAIDs; COX-II, cyclooxygenase II inhibitors; MACEs, major adverse cardiac events

## Discussion

Our results show that in the first 6 months, AS patients treated with NSAIDs did have a non-trivial risk of CVD among those who were non-frequent users. The risk tended to decline with long-term use. In frequent NSAID users, there was no significant risk of CVD, and interestingly, there was a trend showing that the longer the use was, the lower the risk was. Even more, long-term frequent use of COX II had a strong protective effect. The robustness of this finding was supported by the consistency of the results across several pre-specified analyses.

The conclusion that NSAIDs have the potential to increase the occurrence of CVD is based on three clinical trials and epidemiological studies. In the VIGOR study, the COX II inhibitor rofecoxib had a higher incidence of CVD compared with traditional NSAIDs, although in another trial, the CLASS study, celecoxib did not show the same trend. Unfortunately, the CVD risk of COX II inhibitors was further confirmed in two subsequent studies: the APC trial [8] and the APPROVE trial [9]. In these trials, compared with placebo, COX II inhibitors increased CVD risk in patients with a history of colon adenoma. Furthermore, the data showed that the higher the dose was, the higher the risk was. Later epidemiological studies [11] indicated that not only COX II inhibitors but also traditional NSAIDs have the potential to increase CVD risk. Compared with patients with autoimmune diseases, the patients recruited in the above trials were patients with colon adenoma, which is not an inflammatory disease. Additionally, those epidemiological studies examined cardiovascular outcomes in the general population. Few data on NSAIDs’ association with CVD risk within cohorts of patients with autoimmune diseases have been evaluated. Naproxen has been observed to have a cardio protective effect on patients with RA [28,29]. However, the cited reports were full of confounding factors (for example, the authors did not adjust for the drugs used, such as disease-modifying anti-rheumatic drugs (DMARDs), and there was considerable between-study heterogeneity. In a recent meta-analysis, no increased risk was associated with NSAIDs in patients with joint disease [30]. Again, between-study heterogeneity was high. Moreover, the event number was low, and most trials did not include cardiovascular events as the primary outcome, so it is not clear how ascertainment of events was performed in each trial. In an inflammatory polyarthritis cohort study, Goodson et al found that patients exposed to NSAIDs had a 2.5-fold reduction in cardiovascular death compared with non-users [31]. The limitations of their study were 1) the high heterogeneity of the patient population and 2) potential inaccuracy in assessing NSAID exposure. In a recent report from Denmark, Linhardsen et al. observed that the CVD risk associated with NSAID use is lower in RA patients than in patients without RA. Again, the limitation in this study is that the authors ignored the drugs used by the RA patients, which might have had an additional immunomodulatory effect [32].

The strength of the current study is that compared with previous studies, which derived their conclusions from patients with high heterogeneity, we specifically chose patients with AS as the study population to investigate the impact of NSAID use on the risk of cardiovascular events. One reason that we chose patients with AS is that in contrast to use in patients with RA, the use of NSAIDs in the AS patient population is very heterogeneous, so we could compare frequent users with non-frequent users. For RA patients, NSAIDs are usually indispensable. A second reason is that DMARDs (both synthetic and biologic) and steroids are seldom prescribed for the AS population, making the analysis more simple and straightforward.

Recently, interesting observations from clinical studies on patients with AS revealed that NSAIDs could potentially defer radiographic progression [33,34]. Compared with on-demand users, regular NSAID users had less radiographic progression. Meanwhile, patients with high inflammatory marker levels benefitted more from continuous NSAID use. Another observation from a German cohort of patients with ankylosing spondylitis was similar, associating high NSAID intake over 2 years with less mSASSS progression [35]. Based on these solid results, Nigil et al. suggested long-term regular use of NSAIDs among young AS patients without cardiovascular risk to retard radiographic progression, but for older patients, CVD risk should still be considered [36].

In this study we excluded 366 patients who had prior CVD before the diagnosis of AS. This exclusion criteria might cause a potential bias since these patients might have a delayed diagnosis of AS. We had done further analysis which included these 366 patients. The result was similar to the previous analysis. NSAIDs did increase CVD risk in non-frequent users in first 3 months. For frequent users, there was no significant risk of CVD (S3 Table). This study had certain limitations. First, using the data bank, we could not identify which patients had a higher inflammatory status. Inflammatory status might have been a confounding factor. However, theoretically, a higher inflammatory status may cause patients to be prescribed more NSAIDs to relieve symptoms. Second, we were unable to determine real compliance with the drugs prescribed. Third, several of the patients may have taken alternative medicine or may have even bought NSAIDs over the counter by themselves, and these data could not be traced in the database. However, patients only have to pay very low physician fee and one tenth of the drug price for each clinic visit. Total cost will be much lower than they buy the drugs in pharmacy stores. We considered that very low percentage of our patients (especially for those patients with chronic diseases such as AS) got their NSAIDs over the counter. Fourth, it is possible that patients with high CVD risk were channeled away from NSAIDs. This could cause the favorable CVD outcomes observed in NSAID users. Fifth, compared with randomized trials and cohort studies, the level of evidence derived from a case-control study is considered to be lower because the study design is subject to many biases, including in case and control selection, confounding adjustment, and outcome measures. Although we have adjusted for several confounding factors, a major limitation is that unknown confounders may remain, resulting from misclassification of variables or unmeasured variables. Additionally, it seemed that patients were quite old in our database. There are two possibilities: 1) due to delayed diagnosis. In Taiwan, in average, male AS had delayed diagnosis for 5 years, female AS had delayed diagnosis for 10 years. 2): an AS patient might have been ill for years when his data first appeared in the database.

## Conclusion

The results of this study provided data from our cohort for clinicians and patients to judge the risks and benefits of medication when long-term NSAID use is necessary to relieve painful arthritis. Although our data showed that there were no increased risks of CVD in long-term NSAID users. Individual patient must balance the risk/benefit when long-term use is considered. Of course, we need more studies of other autoimmune/auto-inflammatory diseases to determine whether they have similar results as our study. Studies designed both prospectively and retrospectively may help to elucidate the impact of the long-term use of NSAIDs.

## Supporting Information

S1 Table Risk of MACEs associated with NSAIDs in patients with AS stratified by frequency of exposure and types of NSAIDs adjust for Charlson comorbility index and drugs. (DOCX)

S2 Table Risk of stroke associated with NSAIDs in patients with AS stratified by frequency of exposure and types of NSAIDs adjust for Charlson comorbility index and drugs. (DOCX)

S3 Table Risk of all types of cardiovascular diseases associated with NSAIDs in all patients with AS stratified by frequency of exposure and types of NSAIDs adjust for Charlson comorbility index. (DOC)
