# Supplementary material for: Long-Term Frequent Use of Non-Steroidal Anti-Inflammatory Drugs Might Protect Patients with Ankylosing Spondylitis from Cardiovascular Diseases: A Nationwide Case-Control Study
Source: PLoS One. 2015 May 13;10(5):e0126347. doi: 10.1371/journal.pone.0126347 (PMC4430238; doi:10.1371/journal.pone.0126347)
Supplement: S1 Table — (DOCX) [file pone.0126347.s001.docx]

**S1 Table.** **Risk of MACEs associated with NSAIDs in patients with AS stratified by frequency of exposure and types of NSAIDs adjust for Charlson comorbility index and drugs.**

| **Total NSAIDs** | | | | |  | **COX-II** | | | | |  | **Non-selective NSAIDs** | | | | |
| --- | --- | --- | --- | --- | --- | --- | --- | --- | --- | --- | --- | --- | --- | --- | --- | --- |
|  |  | OR | 95%CI | P-value |  |  |  | OR | 95%CI | P-value |  |  |  | OR | 95%CI | P-value |
| 3 months | Non-user | 1 | - | - |  | 3 months | Non-user | 1 | - | - |  | 3 months | Non-user | 1 | - | - |
|  | <80% | 1.24 | 0.86-1.79 | 0.2559 |  |  | <80% | 1.31 | 0.47-3.64 | 0.6108 |  |  | <80% | 1.36 | 0.95-1.95 | 0.0964 |
|  | ≥80% | 0.54 | 0.22-1.35 | 0.1887 |  |  | ≥80% | 0.4 | 0.05-3.16 | 0.3862 |  |  | ≥80% | 0.68 | 0.18-2.57 | 0.5704 |
| 6 months | Non-user | 1 | - | - |  | 6 months | Non-user | 1 | - | - |  | 6 months | Non-user | 1 | - | - |
|  | <80% | 1.11 | 0.75-1.63 | 0.6026 |  |  | <80% | 1.06 | 0.53-2.14 | 0.8659 |  |  | <80% | 1.28 | 0.88-1.86 | 0.1955 |
|  | ≥80% | 0.55 | 0.21-1.47 | 0.2352 |  |  | ≥80% | 0.14 | 0.01-1.85 | 0.1371 |  |  | ≥80% | 0.87 | 0.22-3.53 | 0.8484 |
| 12 months | Non-user | 1 | - | - |  | 12 months | Non-user | 1 | - | - |  | 12 months | Non-user | 1 | - | - |
|  | <80% | 0.72 | 0.45-1.14 | 0.165 |  |  | <80% | 0.74 | 0.41-1.32 | 0.306 |  |  | <80% | 1 | 0.65-1.54 | 0.9853 |
|  | ≥80% | 0.23 | 0.07-0.76 | 0.0159 |  |  | ≥80% | 0.23 | 0.01-5.23 | 0.354 |  |  | ≥80% | 0.25 | 0.05-1.29 | 0.0976 |
| 24 months | Non-user | 1 | - | - |  | 24 months | Non-user | 1 | - | - |  | 24 months | Non-user | 1 | - | - |
|  | <80% | 0.87 | 0.46-1.65 | 0.6683 |  |  | <80% | 0.88 | 0.54-1.44 | 0.6092 |  |  | <80% | 1.02 | 0.57-1.85 | 0.9438 |
|  | ≥80% | 0.26 | 0.06-1.06 | 0.0601 |  |  | ≥80% | 0.72 | 0.07-7.42 | 0.7812 |  |  | ≥80% | 0.35 | 0.04-3.12 | 0.3434 |
| 36 months | Non-user | 1 | - | - |  | 36 months | Non-user | 1 | - | - |  | 36 months | Non- user | 1 | - | - |
|  | <80% | 1.13 | 0.43-3.02 | 0.8019 |  |  | <80% | 0.95 | 0.2-1.46 | 0.8148 |  |  | <80% | 1.55 | 0.65-3.39 | 0.3247 |
|  | ≥80% | 0.32 | 0.06-1.57 | 0.1578 |  |  | ≥80% | 1.9 | 0.09-38.76 | 0.6777 |  |  | ≥80% | 1.19 | 0.12-12.03 | 0.8819 |

Abbreviation: Abbreviation: NSAIDs, non-steroidal anti-inflammatory drugs; Total NSAID, include COX-II inhibitors and non-selective NSAIDs; COX-II, cyclooxygenase II inhibitors; MACEs, major adverse cardiac events
