# Supplementary material for: Long-Term Frequent Use of Non-Steroidal Anti-Inflammatory Drugs Might Protect Patients with Ankylosing Spondylitis from Cardiovascular Diseases: A Nationwide Case-Control Study
Source: PLoS One. 2015 May 13;10(5):e0126347. doi: 10.1371/journal.pone.0126347 (PMC4430238; doi:10.1371/journal.pone.0126347)
Supplement: S2 Table — (DOCX) [file pone.0126347.s002.docx]

**S2 Table.** **Risk of stroke associated with NSAIDs in patients with AS stratified by frequency of exposure and types of NSAIDs adjust for Charlson comorbility index and drugs.**

| **Total NSAIDs** | | | | | | |  | **COX-II** | | | | |  | **Non-selective NSAIDs** | | | | |
| --- | --- | --- | --- | --- | --- | --- | --- | --- | --- | --- | --- | --- | --- | --- | --- | --- | --- | --- |
|  |  | | OR | | 95%CI | P-value |  |  |  | OR | 95%CI | P-value |  |  |  | OR | 95%CI | P-value |
| 3 months | | Non-user | | 1 | - | - |  | 3 months | Non-user | 1 | - | - |  | 3 months | Non-user | 1 | - | - |
|  | | <80% | | 1.31 | 0.88-1.95 | 0.1894 |  |  | <80% | 1.08 | 0.42-2.77 | 0.877 |  |  | <80% | 1.36 | 0.92-2.02 | 0.1251 |
|  | | ≥80% | | 0.93 | 0.36-2.40 | 0.8763 |  |  | ≥80% | 1.25 | 0.23-6.84 | 0.7953 |  |  | ≥80% | 0.32 | 0.05-1.97 | 0.2168 |
| 6 months | | Non-user | | 1 | - | - |  | 6 months | Non-user | 1 | - | - |  | 6 months | Non-user | 1 | - | - |
|  | | <80% | | 1.13 | 0.74-1.71 | 0.5824 |  |  | <80% | 1.62 | 0.78-3.34 | 0.1946 |  |  | <80% | 1.23 | 0.82-1.85 | 0.3241 |
|  | | ≥80% | | 0.94 | 0.36-2.42 | 0.8965 |  |  | ≥80% | 0.63 | 0.09-4.64 | 0.6475 |  |  | ≥80% | 0.92 | 0.19-4.49 | 0.9151 |
| 12 months | | Non-user | | 1 | - | - |  | 12 months | Non-user | 1 | - | - |  | 12 months | Non-user | 1 | - | - |
|  | | <80% | | 0.88 | 0.53-1.45 | 0.603 |  |  | <80% | 1.16 | 0.63-2.14 | 0.626 |  |  | <80% | 1.1 | 0.68-1.78 | 0.6872 |
|  | | ≥80% | | 0.3 | 0.09-0.97 | 0.045 |  |  | ≥80% | 0.36 | 0.03-3.89 | 0.3965 |  |  | ≥80% | 0.34 | 0.05-2.10 | 0.2437 |
| 24 months | | Non-user | | 1 | - | - |  | 24 months | Non-user | 1 | - | - |  | 24 months | Non-user | 1 | - | - |
|  | | <80% | | 0.95 | 0.46-1.97 | 0.8928 |  |  | <80% | 1.25 | 0.73-2.12 | 0.4142 |  |  | <80% | 0.92 | 0.47-1.81 | 0.8052 |
|  | | ≥80% | | 0.57 | 0.13-2.49 | 0.4551 |  |  | ≥80% | 0.53 | 0.04-7.84 | 0.6468 |  |  | ≥80% | 0.84 | 0.06-12.62 | 0.8969 |
| 36 months | | Non-user | | 1 | - | - |  | 36 months | Non-user | 1 | - | - |  | 36 months | Non- user | 1 | - | - |
|  | | <80% | | 1 | 0.32-3.11 | 0.9993 |  |  | <80% | 1.17 | 0.73-1.87 | 0.5068 |  |  | <80% | 1.39 | 0.51-3.81 | 0.5239 |
|  | | ≥80% | | 0.4 | 0.07-2.18 | 0.2878 |  |  | ≥80% | 1.95 | 0.09-41.26 | 0.6677 |  |  | ≥80% | 1.74 | 0.17-18.20 | 0.6441 |

Abbreviation: NSAIDs, non-steroidal anti-inflammatory drugs; Total NSAID, include COX-II inhibitors and non-selective NSAIDs; COX-II, cyclooxygenase II inhibitors; MACEs, major adverse cardiac events
