# Supplementary material for: Long-Term Frequent Use of Non-Steroidal Anti-Inflammatory Drugs Might Protect Patients with Ankylosing Spondylitis from Cardiovascular Diseases: A Nationwide Case-Control Study
Source: PLoS One. 2015 May 13;10(5):e0126347. doi: 10.1371/journal.pone.0126347 (PMC4430238; doi:10.1371/journal.pone.0126347)
Supplement: S3 Table — (DOC) [file pone.0126347.s003.doc]

**S3 Table. Risk of all types of cardiovascular diseases associated with NSAIDs in all patients with AS stratified by frequency of exposure and types of NSAIDs adjust for Charlson comorbility index.**

| **Total NSAIDs** | | | | | | |  | **COX-II** | | | | |  | **Non-selective NSAIDs** | | | | |
| --- | --- | --- | --- | --- | --- | --- | --- | --- | --- | --- | --- | --- | --- | --- | --- | --- | --- | --- |
|  |  | | OR | | 95%CI | P-value |  |  |  | OR | 95%CI | P-value |  |  |  | OR | 95%CI | P-value |
| 3 months | | Non-user | | 1 | - | - |  | 3 months | Non-user | 1 | - | - |  | 3 months | Non-user | 1 | - | - |
|  | | <80% | | 1.53 | 1.21-1.94 | 0.0004 |  |  | <80% | 0.82 | 0.47-1.42 | 0.4767 |  |  | <80% | 1.48 | 1.17-1.85 | 0.0009 |
|  | | ≥80% | | 1.11 | 0.69-1.80 | 0.6712 |  |  | ≥80% | 0.62 | 0.23-1.66 | 0.3416 |  |  | ≥80% | 1.20 | 0.61-2.36 | 0.6070 |
| 6 months | | Non-user | | 1 | - | - |  | 6 months | Non-user |  |  |  |  | 6 months | Non-user | 1 | - | - |
|  | | <80% | | 1.28 | 1.00-1.65 | 0.0533 |  |  | <80% | 0.82 | 0.54-1.25 | 0.3601 |  |  | <80% | 1.25 | 0.98-1.58 | 0.0741 |
|  | | ≥80% | | 1.21 | 0.72-2.04 | 0.4806 |  |  | ≥80% | 0.69 | 0.20-2.44 | 0.5627 |  |  | ≥80% | 1.26 | 0.59-2.70 | 0.5491 |
| 12 months | | Non-user | | 1 | - | - |  | 12 months | Non-user |  |  |  |  | 12 months | Non-user | 1 | - | - |
|  | | <80% | | 1.22 | 0.90-1.67 | 0.2065 |  |  | <80% | 1.01 | 0.72-1.43 | 0.9484 |  |  | <80% | 1.22 | 0.92-1.63 | 0.1706 |
|  | | ≥80% | | 1.20 | 0.66-2.18 | 0.5565 |  |  | ≥80% | 0.31 | 0.05-1.89 | 0.2044 |  |  | ≥80% | 1.56 | 0.67-3.63 | 0.3036 |
| 24 months | | Non-user | | 1 | - | - |  | 24 months | Non-user |  |  |  |  | 24 months | Non-user | 1 | - | - |
|  | | <80% | | 1.13 | 0.71-1.81 | 0.6015 |  |  | <80% | 1.01 | 0.75-1.36 | 0.9450 |  |  | <80% | 1.13 | 0.74-1.73 | 0.5682 |
|  | | ≥80% | | 1.46 | 0.72-2.98 | 0.2943 |  |  | ≥80% | 0.11 | 0.01-0.99 | 0.0491 |  |  | ≥80% | 1.76 | 0.69-4.50 | 0.2368 |
| 36 months | | Non-user | | 1 | - | - |  | 36 months | Non-user |  |  |  |  | 36 months | Non- user | 1 | - | - |
|  | | <80% | | 0.97 | 0.50-1.90 | 0.9298 |  |  | <80% | 1.13 | 0.86-1.49 | 0.3770 |  |  | <80% | 1.10 | 0.61-1.98 | 0.7640 |
|  | | ≥80% | | 0.74 | 0.29-1.91 | 0.5347 |  |  | ≥80% | 0.13 | 0.01-1.22 | 0.0744 |  |  | ≥80% | 1.16 | 0.33-4.04 | 0.8176 |

Abbreviation: NSAIDs, non-steroidal anti-inflammatory drugs; Total NSAID, include COX-II inhibitors and non-selective NSAIDs; COX-II, cyclooxygenase II inhibitors; MACEs, major adverse cardiac events
